# Supplementary material for: Improvement of Biophysical Properties and Affinity of a Human Anti-L1CAM Therapeutic Antibody through Antibody Engineering Based on Computational Methods
Source: Int J Mol Sci. 2021 Jun 22;22(13):6696. doi: 10.3390/ijms22136696 (PMC8268072; doi:10.3390/ijms22136696)
Supplement: Supplementary file 1 [file ijms-22-06696-s001.zip › ijms-1262001-supplementary.pdf]

International Journal of
Molecular Sciences

MDPI

Supplementary Figures

Variant combinations

Variant Name	Light Chain Name	Heavy Chain Name	Comment
Ab417	Ab417_L	Ab417_H	Parental
Ab417_var1	Ab417_L_1	Ab417_H	Light chain FR2 charge distribution corrected
Ab417_var2	Ab417_L_2	Ab417_H_1	Light chain FR2 charge distribution corrected, Threonine at L:46, PTMs removed on heavy chain
Ab417_var3	Ab417_L_3	Ab417_H_1	Light chain FR2 charge distribution corrected, Valine at L:46, PTMs removed on heavy chain
Ab417_var4	Ab417_L_4	Ab417_H_2	Reduced number of positive charges on light and heavy chains
Ab417_var5	Ab417_L_5	Ab417_H_2	Reduced number of positive charges, Threonine at L:46
Ab417_var6	Ab417_L_5	Ab417_H_3	Reduced number of positive charges, Threonine at L:46, PTMs removed on heavy chain
Ab417_var7	Ab417_L_6	Ab417_H_2	Reduced number of positive charges, Valine at L:46
Ab417_var8	Ab417_L_6	Ab417_H_3	Reduced number of positive charges, Valine at L:46, PTMs removed on heavy chain
Ab417_var9	Ab417_L_7	Ab417_H	Reduced surface hydrophobicity in light chain CDRs, Parental heavy chain
Ab417_var10	Ab417_L_8	Ab417_H_2	Reduced positive charges, CDR surface hydrophobicity, L:S46T
Ab417_var11	Ab417_L_8	Ab417_H_3	Reduced positive charges, CDR surface hydrophobicity, L:S46T, PTM removal
Ab417_var12	Ab417_L_9	Ab417_H_2	Reduced positive charges, CDR surface hydrophobicity, L:S46V
Ab417_var13	Ab417_L_9	Ab417_H_3	Reduced positive charges, CDR surface hydrophobicity, L:S46V, PTM removal
Ab417_var14	Ab417_L	Ab417_H_4	CDR H3 substitution H:P110V with Parental light chain
Ab417_var15	Ab417_L	Ab417_H_5	CDR H3 substitution H:P110I with Parental light chain
Ab417_var16	Ab417_L_8	Ab417_H_6	Reduced positive charges, CDR surface hydrophobicity, L:S46T, PTM removal H:P110V
Ab417_var17	Ab417_L_8	Ab417_H_7	Reduced positive charges, CDR surface hydrophobicity, L:S46T, PTM removal, H:P110V
Ab417_var18	Ab417_L_9	Ab417_H_6	Reduced positive charges, CDR surface hydrophobicity, L:S46V, PTM removal, H:P110I
Ab417_var19	Ab417_L_9	Ab417_H_7	Reduced positive charges, CDR surface hydrophobicity, L:S46V, PTM removal, H:P110I
Each variant is composed of two chains (light and heavy). The same chain can appear in multiple variants.			

Figure S1. Variant combinations of Ab417. The final substitutions were combined into nine engineered light chains and seven engineered heavy chains with reduced aggregation propensity. From the combinations of the heavy chain and light chain variants, 19 antibody variants were designed.

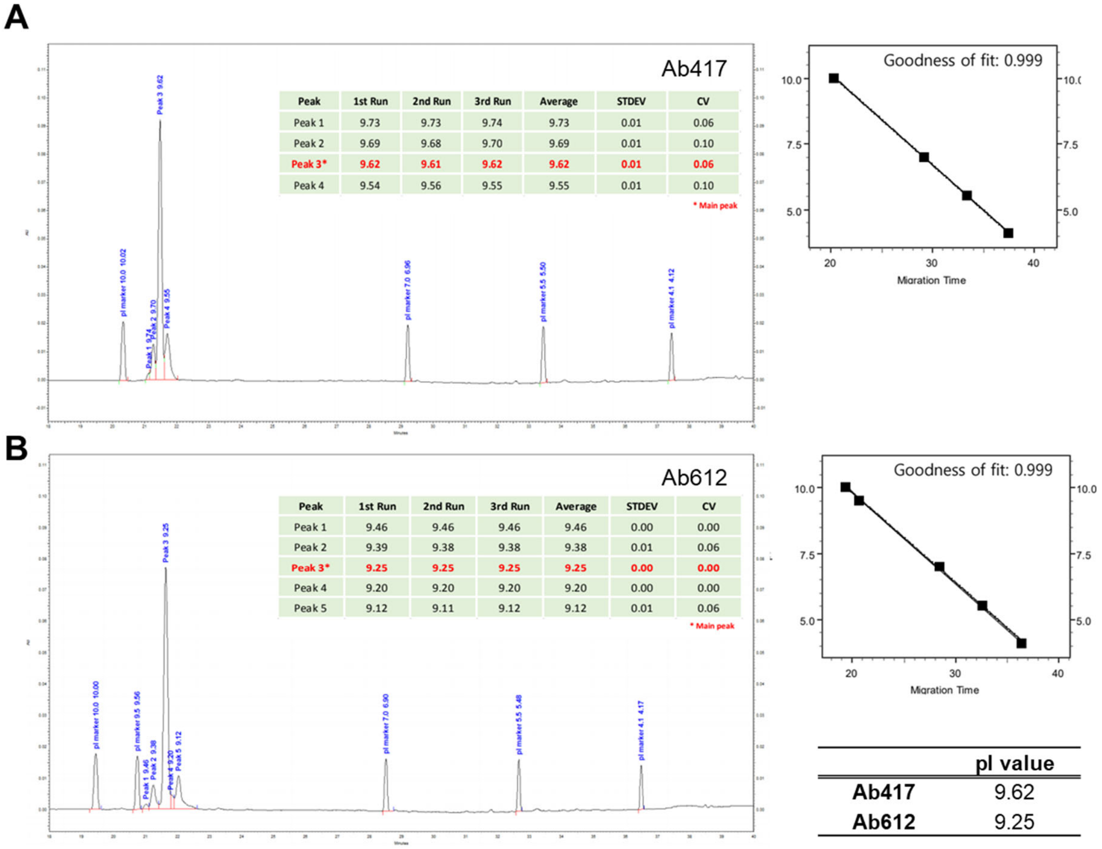

Figure S2. pI value of Ab417 and Ab612 using cIEF. cIEF was performed by SCIEX PA800 plus instrument with neutral coated capillary. All experiments were performed in triplicate. The pI values of the sample were calculated using qualitative analysis of a 32Karat software.
